# Serum miR-122-5p and miR-206 expression: non-invasive prognostic biomarkers for renal cell carcinoma

**DOI:** 10.1186/s13148-018-0444-9

**Published:** 2018-01-23

**Authors:** Frauke G. Heinemann, Yuri Tolkach, Mario Deng, Doris Schmidt, Sven Perner, Glen Kristiansen, Stefan C. Müller, Jörg Ellinger

**Affiliations:** 1 Department of Urology, University Hospital Bonn, Bonn, Germany; 2 Institute of Pathology, University Hospital Bonn, Bonn, Germany; 3 Institute of Pathology, Campus Luebeck, University Hospital Schleswig-Holstein, Luebeck, Germany

**Keywords:** Renal cell carcinoma, miR-122-5p, miR-206, Biomarker, Serum

## Abstract

**Background:**

MicroRNAs (miRNA) play a relevant role in carcinogenesis, cancer progression, invasion, and metastasis. Thus, they can serve as diagnostic/prognostic biomarkers. The knowledge on circulating miRNAs for clear cell renal cell carcinomas (ccRCC) is limited. Our study was designed to identify novel biomarkers for ccRCC patients.

**Results:**

The serum small RNA expression profile was determined in 18 ccRCC and 8 patients with benign renal tumors (BRT) using small RNA sequencing. We detected 29 differentially expressed miRNAs (17 upregulated and 12 downregulated in ccRCC) in the expression profiling cohort. Based on the expression levels, we next validated serum miR-122-5p, miR-193a-5p, and miR-206 levels in an independent cohort (68 ccRCC, 47 BRT, and 28 healthy individuals) using quantitative real-time PCR. Serum expression levels of miR-122-5p and miR-206 were significantly decreased in ccRCC compared to healthy individuals. Both miRNAs were circulating at similar levels in ccRCC and BRT patients. miR-193a-5p expression levels were not different within the study cohort. High serum miR-122-5p and miR-206 levels were associated with adverse clinicopathological parameters: miR-122-5p levels were correlated with metastatic RCC and grade, and miR-206 with pT-stage and metastasis. Furthermore, high miR-122-5p and miR-206 serum levels were associated with a shorter period of progression-free, cancer-specific, and overall survival in patients with ccRCC.

**Conclusion:**

We identified serum miR-122-5p and miR-206 as novel non-invasive prognostic biomarkers for patients with ccRCC.

**Electronic supplementary material:**

The online version of this article (10.1186/s13148-018-0444-9) contains supplementary material, which is available to authorized users.

## Background

Kidney cancer represents 2–3% of human malignancies and among these about 80% are renal cell carcinomas (RCC) [1]. The most common subtype with over 90% is clear cell renal cell carcinoma (ccRCC) [2]. In 2012, 84.400 new cases of kidney cancer and 34.700 cancer deaths occurred in the European Union [3]. Modern ultrasound and computed tomography technologies enable to diagnose kidney tumors in early stages, many of them are in low stage and grade, and up to one third of these tumors are benign [4]. Imaging modalities do not allow precisely distinguishing between benign and malignant tumors, and thus many patients undergo surgery even for non-cancerous tumors. The availability of a non-invasive biomarker could help to avoid unnecessary surgery. Furthermore, prognostic biomarkers could aid the clinician to choose aggressive or rather conservative therapies. Unfortunately, there is no biomarker for ccRCC established in clinical practice by now.

Small non-coding RNAs, especially miRNA, became an important matter for biomarker researchers. miRNAs regulate important cellular functions like apoptosis and proliferation. Differential expressions of miRNAs have been detected in various cancer entities including ccRCC [5]. Several studies highlighted that miRNA expression levels in ccRCC tissue provide diagnostic and prognostic information [6–8]. miRNAs are also circulating in blood and they are characterized by a remarkable stability against degradation by RNases, pH changes, and freeze/thawing [9]. Thus, circulating miRNAs may serve as non-invasive biomarkers. However, less is known about miRNAs in body fluids of ccRCC patients so far: several studies indicated that serum miRNA expression may be of diagnostic interest [10–12]. It was furthermore shown that analysis of a five-miRNAs-panel improved the diagnostic accuracy [13]. A single study also demonstrated that high plasma miR-221 levels were associated with death by RCC [14]. Notably, most earlier studies compared the expression of circulating miRNAs in ccRCC patients with healthy individuals, and only few information is available regarding their expression in patients with benign renal tumors [15].

Other small non-coding RNAs, like PIWI-interacting RNAs (piRNAs), also moved into focus of biomarker researchers. They harbor gene regulatory functions in nucleus and cytoplasm and block transcriptional activity of mRNA synthesis together with PIWI proteins [16, 17]. Two recent studies reported piRNAs in RCC tissue as prognostic biomarkers [18, 19].

In order to improve the current knowledge on circulating small non-coding RNAs and their diagnostic/prognostic relevance in ccRCC patients, we used small RNA sequencing to identify potential novel biomarkers and validated our findings in an independent cohort of ccRCC patients. We thereby identified miR-122-5p and miR-206 as prognostic biomarkers for patients with ccRCC.

## Methods

### Patients

Serum samples were collected within the framework of the Biobank at the CIO Cologne-Bonn at the Department of Urology at the University Hospital Bonn according to standard operating procedures. Serum samples were obtained from patients who underwent radical or partial nephrectomy for renal tumors between 2006 and 2016. All serum samples were collected before surgery. In addition serum samples from healthy donors were obtained. The clinicopathological parameters are shown in Table 1. Overall, cancer-specific and progression-free survival data were available for all patients in discovery and validation cohort. Blood samples were withdrawn in S-Monovette Serum-Gel tubes with clotting activator (Sarstedt, Nümbrecht, Germany). After centrifugation serum was separated and stored in cryotubes at − 80 °C. All patients gave written informed consent. The study was approved by the ethic committee at the University Hospital Bonn (240/14).

**Table 1:** Clinicopathological parameters of the study cohort

	Discovery cohort		Validation cohort		
	ccRCC	BRT	ccRCC	BRT	CTRL
	*n* = 18 (%)	*n* = 8 (%)	*n* = 68 (%)	*n* = 47 (%)	*n* = 28 (%)
Sex					
Male	12 (66.7)	8 (100)	48 (70.6)	27 (57.4)	15 (53.6)
Female	6 (33.3)	0 (0)	20 (29.4)	20 (42.6)	13 (46.4)
Age					
Mean	69.3	64.6	70.4	65.8	54.6
Min-max	51–85	51–81	45–83	43–88	41–69
Pathological stage					
pT1	8 (44.4)	n.a.	38 (55.9)	n.a.	n.a.
pT2	2 (11.1)	n.a.	6 (8.8)	n.a.	n.a.
pT3	8 (44.4)	n.a.	23 (33.8)	n.a.	n.a.
pT4	0 (0)	n.a.	1 (1.5)	n.a.	n.a.
pN1	2 (11.1)	n.a.	4 (5.9)	n.a.	n.a.
pM1	5 (27.8)	n.a.	11 (16.2)	n.a.	n.a.
Fuhrman Grading					
Grade 1	0 (0)	n.a.	6 (8.8)	n.a.	n.a.
Grade 2	6 (33.3)	n.a.	41 (60.3)	n.a.	n.a.
Grade 3	5 (27.8)	n.a.	14 (20.6)	n.a.	n.a.
Grade 4	6 (33.3)	n.a.	7 (10.3)	n.a.	n.a.

*n.a.* not applicable

### Small RNA sequencing

In order to obtain a small RNA expression profile in serum of ccRCC patients, we performed small RNA sequencing experiments with serum samples from patients with ccRCC (*n* = 18) and benign renal tumors (BRT; *n* = 8). The BRT group consisted of four oncocytoma and four complicated kidney cysts; these patients underwent surgery for the suspicious of malignancy. The experiments were carried out by Biogazelle (Zwijnaarde, Belgium) as a contract service. In brief, serum samples were shipped on dry ice to Biogazelle. The RNA was isolated with the Qiagen miRNeasy Serum/Plasma kit (Hilden, Germany). The NEBNext Small RNA Library Prep Set kit (New England Biolabs, Ipswich, USA) was used for library preparation and the small RNA library pools were then sequenced on an Illumina NextSeq 500 sequencer (San Diego, USA). Sequencing reads were mapped to the reference genome (GRCh38) using the short-read-aligner Bowtie [20]. Genome annotation data from miRBase (release21), Ensemble (release 78), and USCS (assembly hg38), and other small RNA types (e.g., piRNA, sn(o)RNA, rRNA and tRNA fragments) was used to annotate the mapped reads to the mature miRNAs. The miRNA expression data were filtered using a cut-off of four reads and normalized based on the total read count per sample. Each miRNA read count was divided by the total read count in that sample and multiplied by the median of total read count across all samples. After normalization all data were log2-transformed. The raw data of the small RNA sequencing are provided at the Gene Expression Omnibus (GEO) database (record: GSE85699).

### Quantitative real-time PCR

In order to validate the small RNA expression profile, we determined exemplarily the expression of three differentially expressed miRNAs using quantitative real-time PCR. The validation cohort included 68 ccRCC, 47 BRT, and 28 healthy individuals. The BRT group consisted of patients, who underwent renal surgery with histological finding of angiomyolipoma, oncocytoma, and complicated kidney cysts. Total RNA was isolated using the mirVana PARIS Kit (Thermo Fisher Scientific, Waltham, USA) from 400 μl serum. Reverse transcription was performed using the miScript II RT Kit (Qiagen, Hilden, Germany). Additionally, 12 cycles of preamplification were performed with the Qiagen miScript PreAMP PCR Kit using a primer mix compiled of the pre-designed primer assays for the target miRNAs miR-122-5p (MS00003416), miR-193a-5p (MS00008932), and miR-206 (MS00003787) and the endogenous reference genes miR-16 (MS00006517), miR-191-5p (MS00003682), and miR-320a (MS00014707) and a custom designed primer assay for the piR-uc032och.1. The endogenous reference genes mir-16, miR-191-5p, and miR-320a were stably expressed in the previous small RNA sequencing experiment and earlier studies demonstrated their usefulness as reference gene [21–23]. Quantitative real-time PCR was performed with 1 μl preamplified cDNA using the Qiagen miScript SYBR Green PCR Kit on an Applied Biosystems 7900 HT Fast Real-Time PCR System (Thermo Fisher Scientific, Waltham, USA) using the Qiagen miScript primer assays described above. The expression data were analyzed with Qbase + (Biogazelle, Zwijnaarde, Belgium) in the 2-ΔΔCT algorithm, using target specific amplification efficiencies and normalization to the reference genes miR-16, miR-191-5p, and miR-320a. Target genes were scaled to average.

### Statistical analysis

The statistical analysis was performed using IBM SPSS Statistics v24 and R v3.3.3. Mann–Whitney–Wilcoxon test was used to compare the expression in subgroups. ROC-analysis and area under curve (AUC) calculations were used to compare the discrimination between control and ccRCC samples for single miRNA expression variables (pROC-package for R). Logistic regression-based model was used to merge the expression of miR-206 and miR-122-5p into one variable to identify the additional discriminative value of simultaneous expression analysis during ROC/AUC-analysis. Optimized cut-off selection during survival analyses was carried out using cutp-function of survMisc package for R (principle: univariate Cox regression-based consecutive analysis of all available cut-offs in the cohort; cut-off selection is based on the best *p* level < 0.05). A *p* value < 0.05 was considered as statistically significant, and all analyses with *p* values between 0.05 and 0.1 were considered to be a trend to statistical significance.

## Results

### Small RNA expression profile

Small RNA sequencing was performed with 26 serum samples to identify differently expressed miRNAs between patients with ccRCC (*n* = 18) and BRT (*n* = 8). Among 2.588 detectable miRNAs, we observed differential expression of 29 miRNAs (*p* < 0.05): 17 miRNAs were up- and 12 miRNAs were downregulated. Most of these miRNAs have not been described dysregulated for RCC by now (e.g., miR-885-3p, miR-450a-2-3p, miR-483-5p, let-7f-1-3p, miR-193a-5p, miR-18a-3p, miR-1185-1-3p, miR-499a-5p, miR-485-3p, miR-125a-5p, miR-4446-3p). miR-122-5p and miR-206 have been examined in renal cell carcinoma tissue so far. A summary of differentially expressed miRNAs in serum of ccRCC and BRT patients is provided in Additional file 1: Table S1.

We also investigated the serum piRNA levels in addition to the miRNA expression profile: 670 piRNAs were detected in serum, but most of them had very low expression values. Only piRNA uc032och.1 was significantly upregulated in serum of ccRCC patients (log2 FC 1.93, *p* < 0.001; see Additional file 1: Figure S1). We created a Qiagen custom miScript primer assay for this piRNA, but given the small amount of piRNA in each serum sample (counts < 332), it was not possible to amplify the piRNA exponentially. Especially, the BRT group showed very low expression levels with an average count of 11.75 in contrast to RCC serum levels (average count = 72).

### Validation of serum miRNA expression

**Fig. 1:**
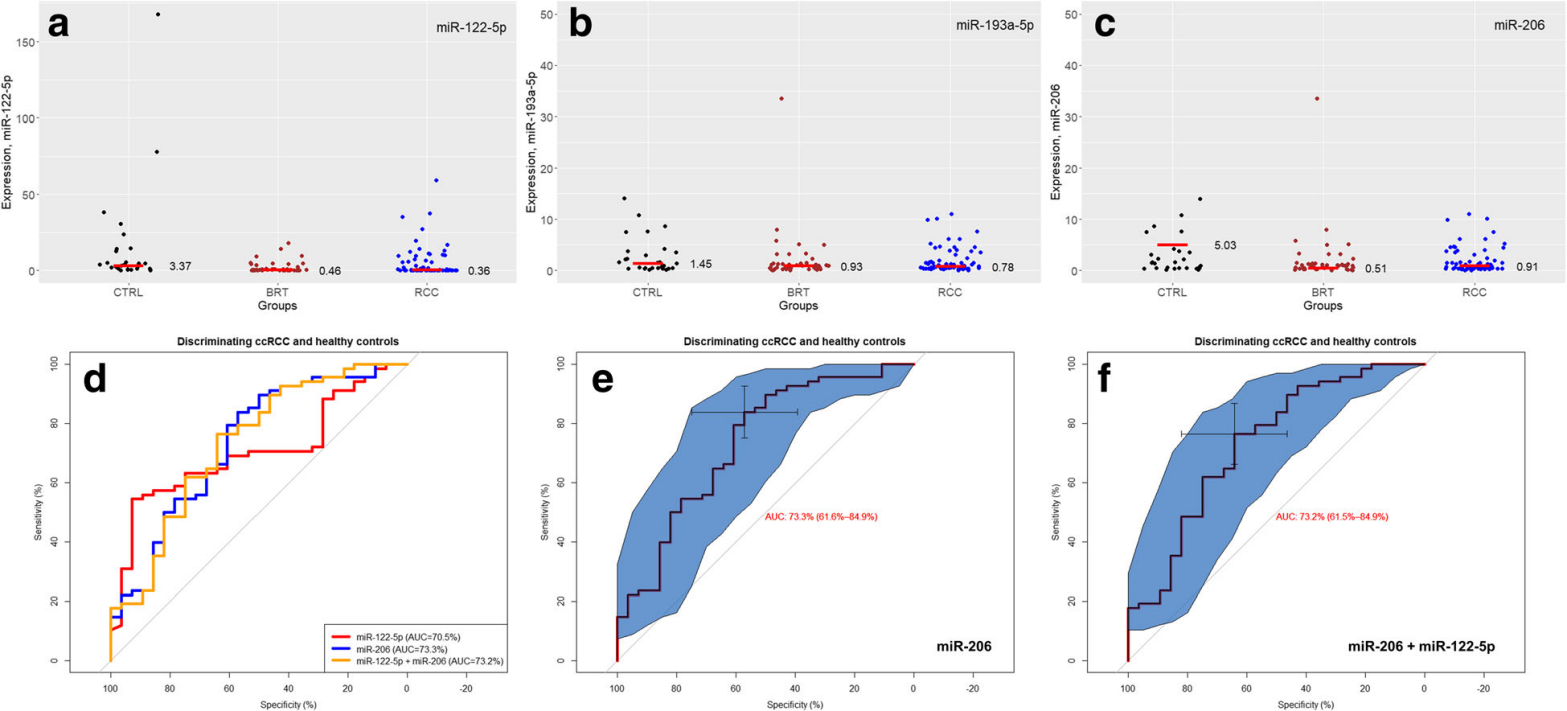
Validation of serum miRNA expression. (**a**–**c**) Serum miRNA expression levels (2-ΔΔCq) were decreased in ccRCC patients compared to healthy individuals for miR-122-5p (**a**) and miR-206 (**c**). Serum miR-193a-5p levels (**b**) were similar in all three subgroups. Red line implies the median value of the expression. **d**–**f** Receiver operator curve (ROC) analyses demonstrate that miR-122-5p and miR-206, as well as combination of both in model allowed discrimination of ccRCC and healthy controls (**d**). Simultaneous analysis of both miRNAs as integral parameter does not allow for better discrimination (**d**, **e**), showing the superiority of miR-206 under miR-193a-5p in terms of discrimination (miR-193a-5p does not provide any additional discriminative capability to that of miR-206). AUC – area under curve with 95% confidence intervals (also outlined as blue space along the ROC-curve). In (**e**, **f**) cross-figure means the best threshold in terms of specificity and sensitivity

To validate the small RNA expression profile, we determined exemplarily the expression of three differentially expressed miRNAs which have not been studied in serum by other researchers yet and which were strongly expressed in serum to allow proper quantification. The independent validation cohort consisted of 143 serum samples including 68 ccRCC, 47 BRT (compiled of 17 oncocytoma, 14 angiomyolipoma and 16 complicated kidney cysts), and 28 healthy individuals. Serum miR-122-5p (log2 fold change − 1.55; *p* = 0.002) and miR-206 (log2 fold change − 1.56; *p* < 0.001) levels were significantly decreased in ccRCC patients compared to healthy individuals (see Fig. 1). miRNA-122-5p was also downregulated in BRT patients compared to healthy individuals (log2 fold change − 2.9; *p* < 0.001). miR-206 was upregulated (log2 fold change 0.36; *p* < 0.001), but this finding is mostly due to three outliers. All miRNAs were circulating at similar levels in BRT and ccRCC patients. Serum miR-193a-5p levels were similar in ccRCC, BRT and control subjects (all *p* > 0.3). To evaluate the usability of these miRNA to serve as serum biomarkers, we performed ROC analyses (Fig. 1 d–f): miR-122-5p, miR-206 and a combination of both could discriminate between ccRCC and healthy controls with an area under the curve (AUC) of up to 0.733 (95% confidence interval: 0.616–0.849) for single miR-206 expression at a specificity of 57.1% and a sensitivity of 83.8% (expression threshold = 4.0).

### Correlation of serum miRNA expression with clinicopathological parameters and survival analyses

**Fig. 2:**
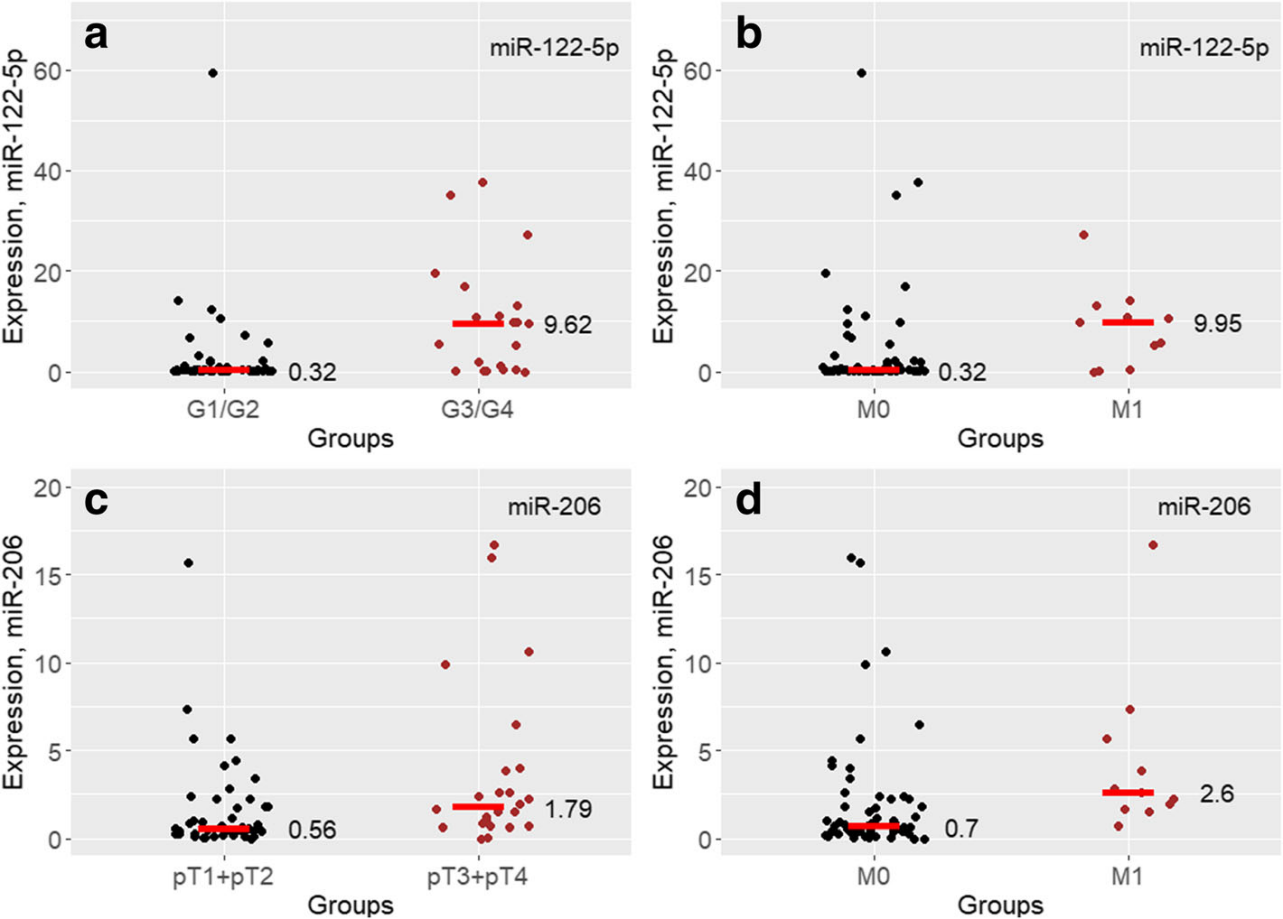
Correlation clinicopathological parameters. miR-122-5p serum levels were significantly increased in advanced Fuhrman Grade (**a**) (*p* = 0.001) and metastasized ccRCCs (**b**) (*p* = 0.044). Serum miR-206 expression was significantly increased in advanced pT-stage (**c**) (*p* = 0.006) and metastasized ccRCC (**d**) (*p* = 0.002). Abbreviations: G1/2, Fuhrman Grade 1 and 2; G3/4, Fuhrman Grade 3 and 4; M0, M-stage 0; M1, M-stage 1; T1/2, T-stage 1 and 2; T3/4, T-stage 3 and 4

We next correlated serum miRNA expression with clinicopathological parameters (see Fig. 2 and Table 2): miR-122-5p levels were significantly increased in metastasized ccRCCs (cM0 vs. cM1; *p* = 0.045) and advanced Fuhrman Grade (G1/2 vs. G3/4; *p* = 0.001). Additionally, we observed a trend of increased miR-122-5p levels in pN1 serum samples (*p* = 0.069). Serum miR-206 expression was significantly increased in advanced pT-stage (pT1/2 vs. pT3/4; *p* = 0.006) and metastasized ccRCC (cM0 vs. cM1; *p* = 0.002) and in advanced Fuhrman Grade as a trend (G1/2 vs. G3/4; *p* = 0.053).

**Table 2:** Correlation of serum miRNA expression with clinicopathological parameters. miR-122-5p and miR-206 serum levels were significantly increased in metastasized ccRCCs. Additionally miR-122-5p expression was significantly increased in advanced Fuhrman Grade and miR-206 in advanced pT-stage

	Number (%)	miR-122-5p (*p* value)	miR-206 (*p* value)
pT-stage		0.145	*0.007*
pT1/2	44 (64.7)		
pT3/4	24 (35.3)		
pN-stage		0.069	0.426
pN0	64 (94.1)		
pN1	4 (5.9)		
pM-stage		*0.045*	*0.003*
pM0	57 (83.8)		
pM1	11 (16.2)		
Fuhrman Grading		*0.001*	0.053
G1/2	47 (69.1)		
G3/4	21 (30.9)		

*Abbreviations T1/2* pT-stage 1 and 2, *T3/4* pT-stage 3 and 4, *G1/2* Fuhrman Grade 1 and 2, *G3/4* Fuhrman Grade 3 and 4

As a next step, we compared the serum expression in advanced clear cell renal cell carcinomas in comparison to the healthy control group. Advanced RCC was defined as pT3/4, lymph node or distant metastasis or Fuhrman Grade 3 or 4. Serum miR-206 expression was significantly increased in advanced ccRCC (log2 fold change − 1.07, *p* = 0.03), whereas miR-122-5p levels were circulating at similar levels.

**Fig. 3:**
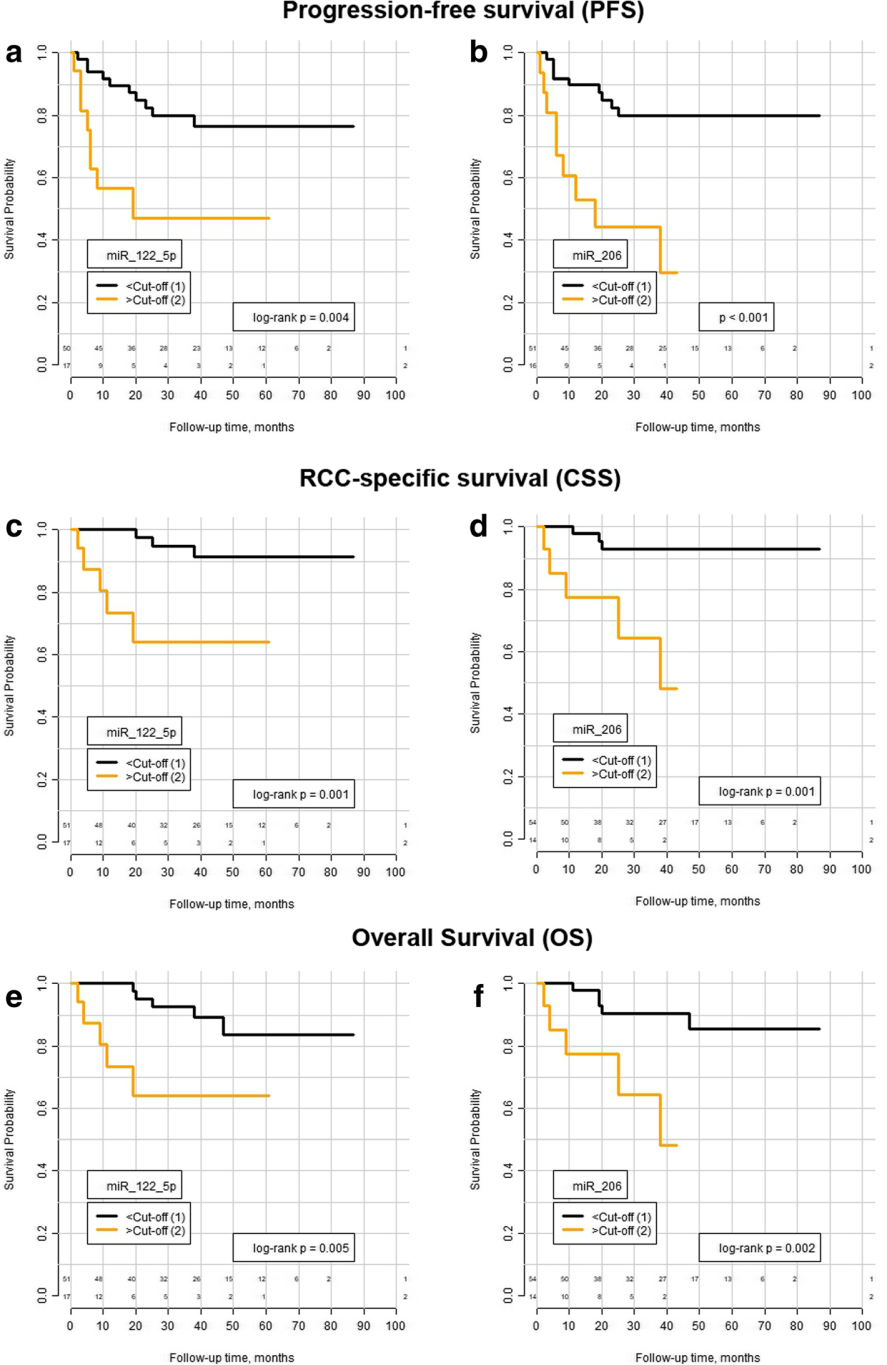
Kaplan Meier estimates. High miR-122-5p and miR-206 serum levels were associated with a shorter period of overall (OS) (**a**, **b**), cancer-specific (CSS) (**c**, **d**), and progression-free survival (PFS) (**e**, **f**)

Univariate cox regression analysis demonstrated that increased miR-122-5p and miR-206 serum levels were correlated (all *p* < 0.005) with a shorter period of progression-free, cancer-specific, and overall survival (see Table 3 for details). Kaplan–Meier visualizes the prognostic relevance of miR-122-5p and miR-206 expression for RCC patient survival (see Fig. 3).

**Table 3:** Univariate Cox regression analyses: miR-122-5p and miR-206 serum levels are correlated with significantly shorter survival periods

hsa-miR-122-5p				
	Cut-off	HR	95% CI	*p* value
Overall survival	5.89	5.11	1.456–17.93	0.010
Cancer-specific survival	5.89	8.146	1.915–34.66	0.004
Progression-free survival	5.89	3.632	1.416–9.318	0.007
hsa-miR-206				
	Cut-off	HR	95% CI	*p* value
Overall survival	2.787	6.037	1.614–22.58	0.007
Cancer-specific survival	2.787	8.15	1.938–34.27	0.004
Progression-free survival	2.382	4.98	1.952–12.7	< 0.001

*Abbreviations*: *HR* hazard ratio, *95%CI* 95% confidence interval

From a statistical point of view, the most evaluable endpoint for multivariate Cox regression analysis in our cohort was progression-free survival (number of patients with complete data 67, number of progression events 18). In a number of models with inclusion of both miRNAs and relevant clinicopathological variables (Table 4), we have shown an independent prognostic value of miR-206 expression in serum, whereas expression of miR-122-5p failed to provide this information. The analysis with regard to other endpoints (cancer-specific and overall survival) is shown in Additional file 1: Table S2 and Additional file 1: Table S3, but it should be noticed that the small cohort size/number of events limits the statistical power.

**Table 4:** Univariate and multivariate Cox regression analysis for miR-122-5p and miR-206 serum levels with progression-free survival as endpoint

	Univariate Analysis			Model 1			Model 2			Model 3			Model 4		
	HR	(95% CI)	*p* value	HR	(95% CI)	*p* value	HR	(95% CI)	*p* value	HR	(95% CI)	*p* value	HR	(95% CI)	*p* value
miR-122-5p, >cut-off vs. <cut-off	3.63	1.42–9.32	0.007	2.11	0.73–6.11	0.169				2.34	0.77–7.11	0.132	1.62	0.45–5.84	0.455
miR-206, >cut-off vs. <cut-off	4.98	1.95–12.7	< 0.001	3.67	1.29–10.51	0.015	3.46	1.13–10.68	0.030				2.94	0.86–10.04	0.084
pT-stage, pT3–4 vs. pT1–2	4.14	1.59–10.79	0.004				1.85	0.58–5.92	0.300	2.68	0.88–8.21	0.082	1.94	0.60–6.29	0.269
pN-stage, pN1 vs. pN0	6.85	1.45–32.47	0.015				4.01	0.68–23.72	0.125	2.72	0.49–15.11	0.252	4.40	0.72–27.01	0.109
M-stage, cM1 vs. cM0	3.61	1.25–10.42	0.018				1.49	0.44–5.01	0.521	1.18	0.34–3.88	0.787	1.40	0.42–4.72	0.583
Fuhrman-Grading, G3 + 4 vs. G1 + 2	4.08	1.58–10.55	0.004				1.48	0.43–5.18	0.536	1.65	0.48–5.67	0.426	1.18	0.29–4.86	0.816

Number of patients in analysis - 67, number of events (progression) - 18

## Discussion

Despite many efforts within the past years, there is no biomarker available for patients with RCC. Circulating miRNAs have been characterized in several studies, but most of them focused on miR-210 [21, 24]. The aim of our study was to identify novel serum miRNAs as non-invasive biomarker for RCC.

Using small RNA sequencing of serum samples from patients with ccRCC and BRT, we discovered 29 differentially expressed miRNAs, thereof 17 upregulated and 12 downregulated. Among these, only miR-99b-5p levels have been examined in serum samples of RCC patients by Lukamowicz-Rajska et al. [25]. They reported increased miR-99b-5p expression levels in RCC tissue in patients with response to tyrosine kinase inhibitor treatment and a long progression-free survival; however, they did not observe a predictive value of circulating miR-99b-5p in serum samples as well.

We exemplarily validated the expression profile by analyzing three miRNAs so far not investigated in serum/plasma of RCC patients. Notably, we observed differential expression of miR-122-5p and miR-206 in ccRCC and healthy subjects, but both miRNAs were circulating at similar levels in ccRCC and BRT patients. In contrast to other studies [14], we did not only compare the serum of patients with ccRCC and a control group with healthy subjects, but also a group of patients with benign renal tumors. This group consists of angiomyolipoma, oncocytoma, and complicated kidney cysts. All patients with benign tumors underwent renal surgery for the suspicious of malignancy. To the best of our knowledge, our study is the first which included this clinically relevant cohort of benign tumors at a larger number.

Even though none of the three miRNAs were successfully validated as diagnostic biomarkers in the independent validation cohort, these miRNAs may be of potential prognostic clinical interest: serum miR-122-5p and miR-206 levels were increased in patients with metastatic disease. Furthermore, miR-122-5p levels were correlated with grade and miR-206 levels with pT-stage. Additionally, we could show that high levels of miR-122-5p and miR-206 were associated with a significantly shorter period of progression-free, cancer-specific, and overall survival. Thus, both miRNA may serve as prognostic parameters. So far, only two studies reported circulating miRNAs in RCC patients to be of potential prognostic relevance: miR-378 levels were increased in patients with advanced pathological stage and correlated with disease-free survival [10] and miR-221 expression was associated with an increased risk of RCC-related death [13].

In the validation cohort, both miR-122-5p and miR-206 were downregulated in serum of ccRCC compared to healthy controls. Interestingly, we determined a shift to increased serum miRNA levels in patients with advanced ccRCC. Serum levels of miR-206 were downregulated in advanced tumors compared to the healthy control group, whereas miR-122-5p expression was circulating at a similar level. This may also explain the finding of miR-122-5p upregulation in the small RNA sequencing experiments, where the proportion of advanced tumors has been slightly higher in discovery cohort.

Increased miR-122-5p expression in ccRCC tissue was described earlier [26–28]. Lian et al. [29] described an increased ability of invasion and migration through activating PI3K/Akt signaling pathway in A498 and 786-O cells overexpressing miR-122. Treatment of CAKI-1 and 786-O cells with a miR-122 inhibitor led to a G0/G1 arrest and overexpression of Spry2, which resulted in activation of the Ras/MAPK pathway and improved tumor cell proliferation [30].

miR-206 was described as tumor suppressor for various tumor entities [31–33], e.g., bladder cancer [34], lung cancer [35], and gastric cancer [36]. In ccRCC, miR-206 is decreased under hypoxia leading to upregulation of VEGF and MET, thereby promoting tumor angiogenesis, invasion, and metastasis [37]. Xiao et al. [38] described that induced upregulation of miR-206 in ACHN and SN_12_PM_6_ cells inhibited cell proliferation and colony forming ability through targeting CDK4, CDK9, and CCND1. Thus, miR-206 is not only a potential biomarker, but also a potential target for the therapy of ccRCC and other cancer entities.

Various studies focused on miRNAs as RCC biomarkers either in tissue or blood samples. Iwamoto et al. [21] and Zhao et al. [39] showed that miR-210 is upregulated in RCC tissues and associated serum samples. Thereby, they were able to point out a connection between modified miRNA expression in cancer tissues and the possibility to detect these miRNAs in serum samples. The cellular function and mechanism of passing into blood circulation of most miRNAs is largely unknown. Our study identifies miR-122-5p and miR-206 as possible biomarkers for renal cell carcinomas, but a direct comparison between corresponding tissue and serum samples is missing so far. Further research is needed on this subject to improve the validity of these serum miRNA biomarkers.

piRNA came increasingly into focus as potential biomarkers. So far, only two studies described a total of six piRNA in renal cell carcinoma tissue with prognostic potential [18, 19]. We were able to detect piRNA uc032och.1 at increased levels in serum of RCC patients via small RNA sequencing. The amount of piRNA seems to be distinctly lower in serum samples than in cancer tissues. Therefore, we were not able to amplify this piRNA using real-time PCR. More stable expressed piRNAs and improved experimental procedures may allow precisely quantifying small amounts of piRNAs in liquid samples and would open an interesting field of research.

Most studies with the focus on liquid biomarkers for tumor entities have used serum samples for their experiments, whereas a minority worked with plasma probes. Wang et al. [40] reported a higher RNA concentration in serum samples than in corresponding plasma probes. The additional amount of RNA may be released from blood cells during the coagulation process, leading to a possible influence of miRNA levels. Bearing this in mind, it is difficult to compare studies based on different raw materials directly. Up to now, only one study described miR-7, miR-221, and miR-222 as potential diagnostic biomarker for renal cell carcinoma [14]. Furthermore, diverging preanalytical parameters, varying amplification techniques, and different normalization approaches make a direct comparison of miRNA studies challenging [15].

Some limitations of our study should also be mentioned: The small RNA sequencing experiment was originally designed to detect serum miRNAs with the ability to discriminate between RCC and benign renal tumors. Instead of being a diagnostic marker, the detected miRNAs turned out to have prognostic potential. Furthermore, angiomyolipomas are relatively safely diagnosed by radiological imaging and these patients usually do not undergo surgery for the suspicion of malignancy in contrast to other patients included in the benign renal tumor group. In addition, our study focused on the analysis of serum samples and the expression of the miRNAs was not validated in renal tissues. Another important step is to confirm our findings in ccRCC tissue samples and investigate the cellular functions of miR-122-5p and miR-206.

## Conclusion

High serum miR-122-5p and miR-206 levels indicate advanced stage/grade and are predictive for a shortened survival following nephrectomy for ccRCC. Thus, miR-122-5p and miR-206 are potential prognostic non-invasive biomarkers for serum of ccRCC patients.

## Additional file

Additional file 1: Supplementary Material. (PDF 233 kb)

## References

[1] Ljungberg B, Bensalah K, Bex A (2016). EAU Guidelines on Renal Cell Carcinoma.

[2] Capitanio U, Cloutier V, Zini L (2009). A critical assessment of the prognostic value of clear cell, papillary and chromophobe histological subtypes in renal cell carcinoma: a population-based study. BJU Int.

[3] Ferlay J, Steliarova-Foucher E, Lortet-Tieulent J (2013). Cancer incidence and mortality patterns in Europe: estimates for 40 countries in 2012. Eur J Cancer.

[4] Sheth S, Scatarige JC, Horton KM (2001). Current concepts in the diagnosis and management of renal cell carcinoma: role of multidetector ct and three-dimensional CT. Radiographics.

[5] Calin GA, Croce CM (2006). MicroRNA signatures in human cancers. Nat Rev Cancer.

[6] Li M, Wang Y, Song Y (2015). MicroRNAs in renal cell carcinoma: a systematic review of clinical implications (review). Oncol Rep.

[7] Gu L, Li H, Chen L (2015). MicroRNAs as prognostic molecular signatures in renal cell carcinoma: a systematic review and meta-analysis. Oncotarget.

[8] Al-Ali BM, Ress AL, Gerger A, Pichler M (2012). MicroRNAs in renal cell carcinoma: implications for pathogenesis, diagnosis, prognosis and therapy. Anticancer Res.

[9] Chen X, Ba Y, Ma L (2008). Characterization of microRNAs in serum: a novel class of biomarkers for diagnosis of cancer and other diseases. Cell Res.

[10] Wulfken L, Moritz R, Ohlmann C (2011). MicroRNAs in renal cell carcinoma: diagnostic implications of serum miR-1233 levels. PLoS One.

[11] Fedorko M, Stanik M, Iliev R (2015). Combination of MiR-378 and MiR-210 serum levels enables sensitive detection of renal cell carcinoma. Int J Mol Sci.

[12] Hauser S, Wulfken LM, Holdenrieder S (2012). Analysis of serum microRNAs (miR-26a-2*, miR-191, miR-337-3p and miR-378) as potential biomarkers in renal cell carcinoma. Cancer Epidemiol.

[13] Wang C, Hu J, Lu M (2015). A panel of five serum miRNAs as a potential diagnostic tool for early-stage renal cell carcinoma. Sci Rep.

[14] Texeira AL, Ferreira M, Silva J (2014). Higher circulating expression levels of miR-221 associated with poor overall survival in renal cell carcinoma patients. Tumor Biol.

[15] Ellinger J, Gevensleben H, Müller SC, Dietrich D (2016). The emerging role of non-coding circulating RNA as a biomarker in renal cell carcinoma. Expert Rev Mol Diagn.

[16] Ng KW, Anderson C, Marshall EA (2016). Piwi-interacting RNAs in cancer: emerging functions and clinical utility. Mol Cancer.

[17] Suzuki R, Honda S, Kirino Y (2012). PIWI expression and function in cancer. Front Genet.

[18] Busch J, Ralla B, Jung M (2015). Piwi-interacting RNAs as novel prognostic markers in clear cell renal cell carcinomas. J Exp Clin Cancer Res.

[19] Li Y, Wu X, Gao H (2015). Piwi-interacting RNAs (piRNAs) are Dysregulated in renal cell carcinoma and associated with tumor metastasis and cancer-specific survival. Mol Med.

[20] Langmead B, Trapnell C, Pop M, Salzberg SL (2009). Ultrafast and memory-efficient alignment of short DNA sequences to the human genome. Genome Biol.

[21] Iwamoto H, Kanda Y, Sejima T (2014). A. Serum miR-210 as a potential biomarker of early clear cell renal cell carcinoma. Int J Oncol.

[22] Wang L, Liu Y, Du L (2015). Identification and validation of reference genes for the detection of serum microRNAs by reverse transcription-quantitative polymerase chain reaction in patients with bladder cancer. Mol Med Rep.

[23] Zheng G, Wang H, Zhang X (2013). Identification and validation of reference genes for qPCR detection of serum microRNAs in colorectal Adenocarcinoma patients. PLoS One.

[24] Texeira AL, Dias F, Gomes M (2014). Circulating biomarkers in renal cell carcinoma: the link between microRNAs and extracellular vesicles, where are we now?. J Kidney Cancer VHL.

[25] Lukamowicz-Rajska M, Mittmann C, Prummer M (2016). MiR-99b-5p expression and response to tyrosine kinase inhibitor treatment in clear cell renal cell carcinoma patients. Oncotarget.

[26] White NMA, Bao TT, Grigull J (2011). miRNA profiling for clear cell renal cell carcinoma: biomarker discovery and identification of potential controls and consequences of miRNA Dysregulation. J Urol.

[27] Jung M, Mollenkopf H-J, Grimm C (2009). MicroRNA profiling of clear cell renal cell cancer identifies a robust signature to define renal malignancy. J Cell Mol Med.

[28] Osanto S, Qin Y, Buermans HP (2012). Genome-wide MicroRNA expression analysis of clear cell renal cell carcinoma by next generation deep sequencing. PLoS One.

[29] Lian J-H, Wang W-H, Wang J-Q (2013). MicroRNA-122 promotes proliferation, invasion and migration of renal cell carcinoma cells through the PI3K/Akt signaling pathway. Asian Pac J Cancer Prev.

[30] Wang Z, Qin C, Zhang J (2017). MiR-122 promotes renal cancer cell proliferation by targeting Sprouty3. Tumor Biol.

[31] Mitchelson KR, Qin WY (2015). Roles of the canonical myomiRs miR-1, −133 and −206 in cell development and disease. World J Biol Chem.

[32] Nohata N, Hanazawa T, Enokida H, Seki N (2012). microRNA-1/133a and microRNA-206/133b clusters: Dysregulation and functional roles in human cancers. Oncotarget.

[33] Novak J, Kruzliak P, Bienertova-Vasku J (2014). MicroRNA-206: a promising Theranostic marker. Theranostics.

[34] Huang B, Zhai W, Hu G (2016). MicroRNA-206 acts as a tumor suppressor in bladder cancer via targeting YRDC. Am J Transl Res.

[35] Wang X, Ling C, Bai Y, Zhao J (2011). MicroRNA-206 is associated with invasion and metastasis of lung cancer. Anat Rec (Hoboken).

[36] Ren J, Huang H-J, Gong Y (2014). MicroRNA-206 suppresses gastric cancer cell growth and metastasis. Cell Biosci.

[37] Müller S, Nowak K (2014). Exploring the miRNA-mRNA regulatory network in clear cell renal cell carcinomas by next-generation sequencing expression profiles. Biomed Res Int.

[38] Xiao H, Xiao W, Cao J (2016). miR-206 functions as a novel cell cycle regulator and tumor suppressor in clear-cell renal cell carcinoma. Cancer Lett.

[39] Zhao A, Li G, Péoc’h M (2013). Serum miR-210 as a novel biomarker for molecular diagnosis of clear cell renal cell carcinoma. Exp Mol Pathol.

[40] Wang K, Yuan Y, Cho J-H (2012). Comparing the MicroRNA Spectrum between serum and plasma. PLoS One.

