# Supplementary material for: Serum miR-122-5p and miR-206 expression: non-invasive prognostic biomarkers for renal cell carcinoma
Source: Clin Epigenetics. 2018 Jan 23;10:11. doi: 10.1186/s13148-018-0444-9 (PMC5781339; doi:10.1186/s13148-018-0444-9)

Table S1. Small RNA Sequencing. Differentially ($p<0.05$) expressed miRNAs in serum of RCC patients.

Upregulated in ccRCC	log2 FC	Downregulated in ccRCC	log2 FC
hsa-miR-122-5p	0.88	hsa-miR-206	-1.31
hsa-miR-885-3p	0.85	hsa-miR-1185-1-3p	-0.72
hsa-miR-193b-5p	0.78	hsa-miR-499a-5p	-0.70
hsa-miR-450a-2-3p	0.75	hsa-miR-485-3p	-0.65
hsa-miR-483-5p	0.62	hsa-miR-125a-5p	-0.61
hsa-let-7f-1-3p	0.60	hsa-miR-4446-3p	-0.59
hsa-miR-193a-5p	0.59	hsa-miR-196b-5p	-0.58
hsa-miR-10b-3p	0.59	hsa-miR-625-3p	-0.53
hsa-miR-18a-3p	0.58	hsa-let-7i-5p	-0.50
hsa-miR-582-3p	0.58	hsa-miR-139-3p	-0.48
hsa-miR-150-3p	0.55	hsa-miR-99b-5p	-0.44
hsa-miR-22-5p	0.54	hsa-miR-1908-5p	-0.40
hsa-miR-194-5p	0.53		
hsa-miR-148a-3p	0.52		
hsa-miR-192-5p	0.46		
hsa-miR-27a-3p	0.43		
hsa-miR-142-5p	0.35		

Table S2. Multivariate Cox regression analysis for miR-122-5p.
Cut-off 5.89. Abbreviations: HR, hazard ratio; 95%CI, 95% confidence interval

	Overall Survival (OS)			Cancer Specific Survival (CSS)			Progression-free Survival (PFS)		
	**HR**	**(95% CI)**	**p-value**	**HR**	**(95% CI)**	**p-value**	**HR**	**(95% CI)**	**p-value**
**miR-122-5p, >cut-off vs <cut-off**	5.04	1.16 - 21.94	0.031	8.61	1.56 - 47.44	0.013	2.34	0.77 - 7.11	0.132
**pT-stage, pT3-4 vs pT1-2**	0.11	0.01 - 1.12	0.063	0.11	0.01 - 1.22	0.072	2.68	0.88 - 8.21	0.082
**pN-stage pN1 vs pN0**	6.49	1.20 - 35.2	0.03	42.26	1.38 - 1289.94	0.032	2.72	0.49 - 15.11	0.252
**M-stage, cM1 vs cM0**	33.53	1.28 - 876.06	0.035	7.36	1.24 - 43.82	0.028	1.18	0.34 - 3.88	0.787
**Fuhrman-Grading, G3+4 vs G1+2**	1.44	0.26 - 7.93	0.672	1.35	0.23 - 8.07	0.74	1.65	0.48 - 5.67	0.426

Table S3. Multivariate Cox regression analysis for miR-206. Cut-off 2.787 for OS and CSS, Cut-off 2.382 for PFS. Abbreviations: HR, hazard ratio; 95%CI, 95% confidence interval

	Overall Survival (OS)			Cancer Specific Survival (CSS)			Progression-free Survival (PFS)		
	**HR**	**(95% CI)**	**p-value**	**HR**	**(95% CI)**	**p-value**	**HR**	**(95% CI)**	**p-value**
**miR-122-5p, >cut-off vs <cut-off**	8.57	1.91 - 38.38	0.005	13.14	2.43 - 70.93	0.003	3.46	1.13 - 10.68	0.030
**pT-stage, pT3-4 vs pT1-2**	0.07	0.01 - 0.82	0.034	0.05	0.003 - 0.78	0.032	1.85	0.58 - 5.92	0.300
**pN-stage pN1 vs pN0**	24.9	1.18 - 525.18	0.039	26.22	1.18 - 582.29	0.038	4.01	0.68 - 23.72	0.125
**M-stage, cM1 vs cM0**	3.8	0.84 - 17.18	0.082	4.62	0.96 - 22.29	0.057	1.49	0.44 - 5.01	0.521
**Fuhrman-Grading, G3+4 vs G1+2**	5.75	1.01 - 32.70	0.048	8.57	1.23 - 59.69	0.030	1.48	0.43 - 5.18	0.536

Figure S1. Serum piRNA levels. piRNA expression values were low in the 26 serum samples (A). piRNA uc032och.1 was significantly upregulated in serum of ccRCC patients (B).

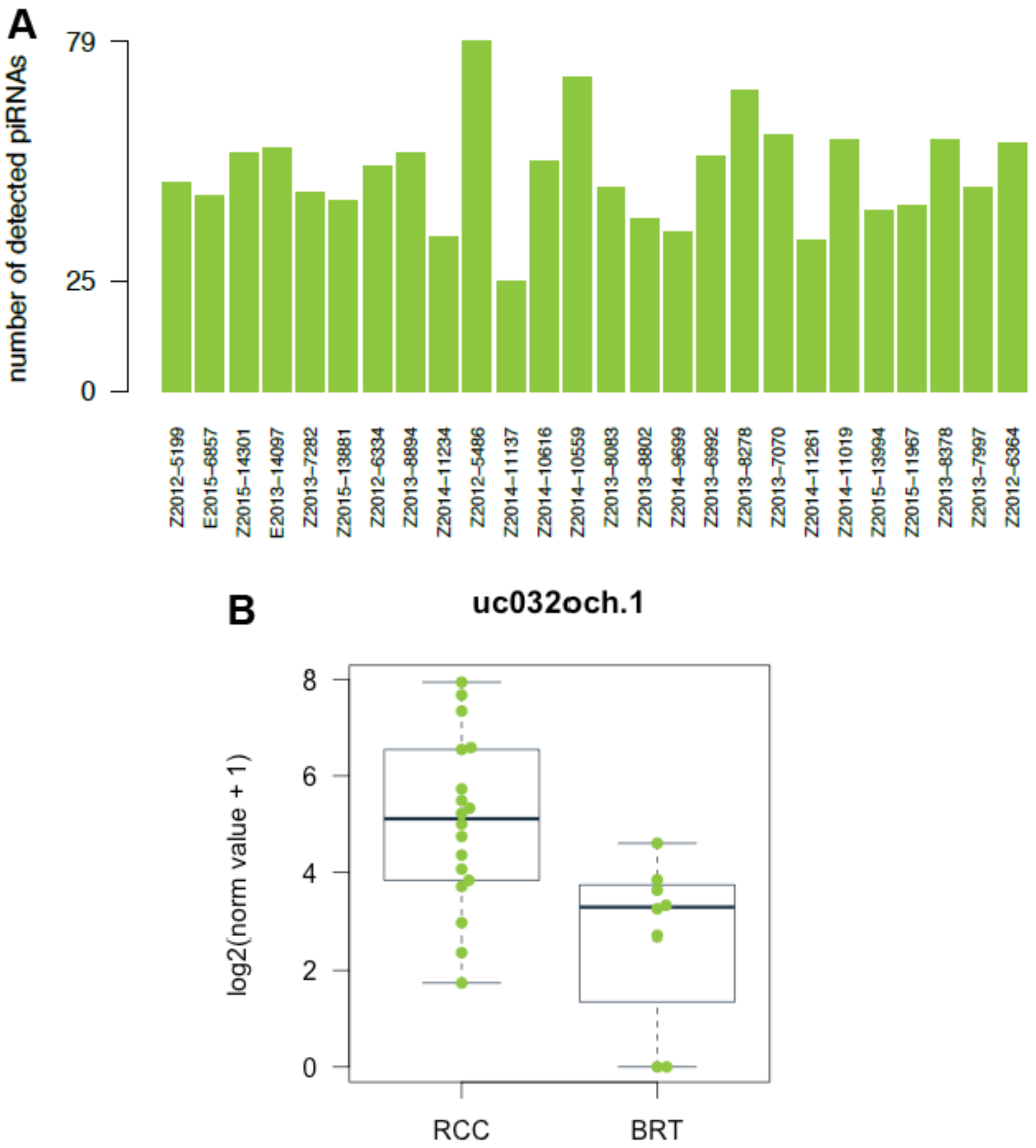

Supplement: Supplementary file 1 — Supplementary Material. (PDF 233 kb) [file 13148_2018_444_MOESM1_ESM.pdf]
